# Mavoglurant in adolescents with fragile X syndrome: analysis of Clinical Global Impression-Improvement source data from a double-blind therapeutic study followed by an open-label, long-term extension study

**DOI:** 10.1186/s11689-015-9134-5

**Published:** 2015-12-15

**Authors:** Donald B. Bailey, Elizabeth Berry-Kravis, Anne Wheeler, Melissa Raspa, Florence Merrien, Javier Ricart, Barbara Koumaras, Gerd Rosenkranz, Mark Tomlinson, Florian von Raison, George Apostol

**Affiliations:** RTI International, Research Triangle Park, Durham, NC USA; Department of Pediatrics, Neurological Sciences, and Biochemistry, Rush University Medical Centre, Chicago, IL 60612 USA; Neuroscience Development, Novartis Pharma AG, Basel, Switzerland; Novartis Pharma, Barcelona, Spain; Neurodegeneration Global Development, Novartis Pharmaceuticals Corporation, East Hanover, NJ USA

**Keywords:** Mavoglurant, AFQ056, Fragile X syndrome, Clinical Global Impression-Improvement, CGI-I

## Abstract

**Background:**

A phase II randomized, placebo-controlled, double-blind study and subsequent open-label extension study evaluated the efficacy, safety, and tolerability of mavoglurant (AFQ056), a selective metabotropic glutamate receptor subtype-5 antagonist, in treating behavioral symptoms in adolescent patients with fragile X syndrome (FXS). A novel method was applied to analyze changes in symptom domains in patients with FXS using the narratives associated with the clinician-rated Clinical Global Impression-Improvement (CGI-I) scale.

**Methods:**

In the core study, patients were randomized to receive mavoglurant (25, 50, or 100 mg BID) or placebo over 12 weeks. In the extension, patients received 100 mg BID mavoglurant (or the highest tolerated dose) for up to 32 months. Global improvement, as a measure of treatment response, was assessed using the CGI-I scale. Investigators assigning CGI-I scores of 1 (very much improved), 2 (much improved), 6 (much worse), or 7 (very much worse) were provided a standard narrative template to collect further information about the changes observed in patients. Investigator feedback was coded and clustered into categories of improvement or worsening to identify potential areas of improvement with mavoglurant. Treatment effect in each category was characterized using the Cochran–Mantel–Haenszel test.

**Results:**

A total of 134 and 103 patients had reached 2 weeks or more of core and extension study treatment, respectively, by the pre-assigned cutoff date for investigator feedback. In the core study, 34 CGI-I scores of 1 or 2 were reported in 28 patients; one patient scored 6. Analysis of the CGI-I narratives did not indicate greater treatment response in patients receiving mavoglurant compared with placebo in any specific improvement domain. There were 54 CGI-I scores of 1 or 2 in 47 patients in the extension study. The most frequently reported categories of improvement were behavior and mood (79.3 and 76.6 % in core and extension studies, respectively), engagement (75.9 and 78.7 %), and communication (69.0 and 61.7 %).

**Conclusions:**

A method was established to capture and categorize FXS symptoms using CGI-I narratives. Although this method did not show benefit of drug over placebo, narratives from investigators were mostly based on parental report and thus do not represent a completely objective alternative assessment.

**Trial registration:**

The studies described are registered at ClinicalTrials.gov with clinical trial identifier numbers NCT01357239 and NCT01433354.

## Background

Fragile X syndrome (FXS) is an X-linked genetic condition associated with an expansion of the trinucleotide CGG repeat within the 5’ untranslated region of the fragile X mental retardation 1 (*FMR1*) gene [1]. It is the most common inherited cause of intellectual disability with a prevalence of 1 in 4000–7000 for males and 1 in 8000–11,000 for females [2, 3]. Individuals with a full mutation frequently present with mild-to-moderate intellectual and learning disabilities.

FXS is associated with a heterogeneous clinical phenotype, which is characterized by cognitive and behavioral impairments, as well as physical features such as macroorchidism (in males), macrocephaly, large prominent ears, hypotonia, long face, flat feet, soft skin, high-arched palate, and hyperextensible joints [4–6]. Female individuals with FXS tend to present with less severe physical and behavioral characteristics than males. Common behavioral symptom categories include anxiety, hyperactivity, aggression, attention deficits, impulsivity, and mood lability, with motor stereotypies, social avoidance, and poor eye contact also often present [5]. In addition, features of autistic spectrum disorder are found in a high proportion of patients with FXS [4–6].

The full mutation leads to methylation of the surrounding nucleotide sequences and hence inhibition of *FMR1* gene transcription [7–9]. The fragile X mental retardation protein (FMRP, the gene product of *FMR1*) is an RNA-binding protein involved in the transport [10] and translational regulation of various dendritic mRNAs at synapses [11]. Absence of FMRP results in defects in synaptic plasticity and enhanced long-term depression (LTD) [12, 13]. On the basis of findings from several studies, it has been proposed that dysregulation of the metabotropic glutamate receptor subtypes 1 and 5 (mGluR1/5) pathways, involved in LTD and synaptic plasticity, contributes to many of the symptoms of FXS [13]. A number of clinical trials have assessed the therapeutic potential of strategies targeting the mGluR5 pathways. The agents investigated include fenobam, RG7090 (R04917523), and mavoglurant (AFQ056) [4, 14, 15].

Mavoglurant, a selective mGluR5 antagonist, has been evaluated for the treatment of behavioral symptoms in patients with FXS. In a phase II, randomized, double-blind, placebo-controlled, two-treatment, two-period, crossover study of 30 male patients with FXS aged 18–35 years, no significant treatment differences were found between mavoglurant and placebo on the primary outcome measure, the Aberrant Behavior Checklist-Community Edition (ABC-C) total score, at day 19 or 20 [15]. Furthermore, no significant effects of mavoglurant on other secondary outcome measures, including the Clinical Global Impression (CGI) scale, Visual Analog Scale (VAS) of behavior, Social Responsiveness Scale-Adult Research Version (SRS-A), and Vineland Adaptive Behavior Scale (VABS), were reported. However, an exploratory analysis suggested improvement in the ABC-C total score (*p* < 0.001), ABC-C stereotypic behavior, hyperactivity, inappropriate speech subscales (*p* < 0.05), CGI-Improvement (CGI-I; *p* < 0.001), Repetitive Behavior Scale-Revised (RBS-R), SRS-A, and VAS (all *p* < 0.05) in a subpopulation of fully methylated males.

Subsequent mavoglurant trials have been completed in adult (aged 18–45 years; NCT01253629) and adolescent (aged 12–17 years; NCT01357239) patients with FXS. These studies were 12-week, phase II, randomized, double-blind, placebo-controlled, parallel-group studies and have been followed up with long-term, open-label extension studies in adults (NCT01348087) and adolescents (NCT01433354). In the double-blind studies, participants were divided into two strata depending on the extent of methylation of their *FMR1* gene. Multiple behavioral outcome measures were implemented across these studies; however, mavoglurant did not demonstrate improvement in behaviors with respect to placebo on either primary or secondary end points (ABC-C using the FXS specific algorithm (ABC-C_FX_), CGI-I, RBS-R, and SRS).

The broad spectrum of phenotypes in FXS makes finding the optimal outcome measures a major challenge. In order to capture a range of potential changes across a number of cognitive and behavioral domains in FXS, trials of pharmaceutical interventions have incorporated multiple end points, including ABC-C and CGI [16]. With the exception of ABC-C, for which a factor analysis specific to FXS has been performed and subscales created on the basis of the FXS factors [17], these outcome measures are not specific to FXS and are used across a range of central nervous system conditions.

Clinical trials involving patients with FXS regularly use the CGI scales as secondary end points in the assessment of treatment response. CGI is a clinician-rated, interview-based assessment that takes into account all available information, including symptoms, behavior, functionality, patient history, and reports from caregivers and other sources [18]. It comprises three components: severity (CGI-S), global improvement (CGI-I), and efficacy (CGI-E) [19]. CGI-S and CGI-I are the most commonly used scales in clinical trials and both are scored on a similar seven-point scale to acquire an overall assessment of a patient’s condition. The CGI-S rates illness severity at the time of assessment (1 = normal, not at all ill; 2 = borderline, mentally ill; 3 = mildly ill; 4 = moderately ill; 5 = markedly ill; 6 = severely ill; and 7 = among the most extremely ill patients), whereas the CGI-I rates change from baseline or from the beginning of treatment (1 = very much improved; 2 = much improved; 3 = minimally improved; 4 = no change; 5 = minimally worse; 6 = much worse; and 7 = very much worse).

These scales were also used as the primary outcome measure in a randomized, double-blind, placebo-controlled trial of minocycline in children and adolescents with FXS [20], in which a marginal but statistically significant (*p* = 0.0173); improvement in the CGI-I was reported with minocycline compared with placebo. However, there are no standardized scoring anchors or interview guidelines for this instrument, and consequently, the CGI-I assessment is vulnerable to inter-rater variability and poor reliability [21, 22]. Furthermore, although the CGI has previously been adapted for bipolar disorder [23], schizophrenia [24], Alzheimer’s disease [25], and depression [26], to date, a version has not been developed specifically for patients with FXS.

The analyses of the overall symptoms in adolescent patients with FXS from a 12-week, phase II, randomized, double-blind, placebo-controlled study (NCT01357239) using the narratives associated with the clinician-rated CGI-I scale are presented. Analysis of the CGI-I narratives from a follow-on open-label extension study (NCT01433354) in adolescents with FXS has also been included in order to describe the reports in the long-term study and to see if the effects become diminished over time, as might be expected if there is a placebo effect.

This analysis was prompted by anecdotal reports regarding clinical improvement received from investigators during the double-blind treatment, relating to symptom categories not captured by the scales used in the study. The intention of this analysis was to investigate the effect of mavoglurant treatment versus placebo in categories generated from analysis of the CGI-I narratives: anxiety, behavior and mood, communication, cognitive/academic, engagement, and functional skills, in adolescent patients with FXS who reached a CGI-I score of 1, 2, 6, or 7 (i.e., patients who were considered much or very much improved or worse) at any time during the study.

## Methods

### Study design

This was a prospective analysis of change in the overall symptoms of FXS from a multicenter, phase II, randomized, placebo-controlled, double-blind, parallel-group study (NCT01357239) with an open-label extension (NCT01433354) in adolescent patients with FXS. The primary results of the double-blind study have been described by Berry-Kravis et al. and the open-label study will also be reported separately.

In the core double-blind study, a protocol amendment was implemented during randomization after approximately 70 patients had been randomized. After assessment of eligibility and a 4-week, single-blind, placebo run-in period, under the original protocol, patients were randomized in a ratio of 1:1:1:1 to one of four treatment arms to receive mavoglurant 25 mg BID (one capsule of 25 mg and one capsule of placebo per intake), 50 mg BID (two capsules of 25 mg per intake), or 100 mg BID (one capsule of 100 mg and one capsule of placebo per intake), or placebo (two capsules of placebo per intake) over a 12-week, double-blind period. Following the protocol amendment, newly enrolled patients were randomized in a ratio of 1:1 to one of two treatment arms to receive mavoglurant 100 mg BID (one capsule of 100 mg and one capsule of placebo per intake) or placebo BID (two capsules of placebo per intake). Patients randomized before the protocol amendment continued on their assigned dose until the end of the double-blind period. The study population was stratified by gender, methylation status, and region during randomization. This study was initiated in May 2011 and completed in January 2014.

The extension was an open-label, flexible-dose, long-term safety study in adolescent patients with FXS who had participated in previous mavoglurant studies. Patients received a starting dose of mavoglurant 25 mg BID, to maintain blinding of the study in which the patients had previously participated, and were incrementally up-titrated to a maximum dose of 100 mg BID or the highest tolerated dose (25, 50, or 75 mg BID). Patients received treatment for up to 32 months but discontinued the treatment when the study was terminated early based on the results of the 12-week double-blind studies in adults (NCT01253629) and adolescents (NCT01357239) which failed to demonstrate efficacy of mavoglurant in the target population and the sponsor’s decision to terminate the program.

The study protocols were reviewed by the Institutional Review Board for each center. The studies were conducted according to the ethical principles of the Declaration of Helsinki. Written informed consent was obtained from each patient’s legal guardian before any assessment was performed.

### Participants

The patients eligible to participate in the core study were adolescent male or female patients aged 12–17 years of age with a diagnosis of FXS confirmed by genetic testing results (>200 CGG repeats). Patients were also required to have a CGI-S score of ≥4 (moderately ill), a score of >20 on the ABC-C total scale, and an intelligence quotient of <70 as measured by the Leiter International Performance Scale-Revised. Inclusion in the open-label extension study required participation in previous mavoglurant studies that had included adolescent patients with FXS up to 18 years of age, provided they were at least 12 years of age at the time of enrollment into the extension study. Exclusion criteria are presented in detail in the core study paper (Berry-Kravis et al 2015) [27]. In addition, patients were not eligible for enrollment into the extension study if they had discontinued from another mavoglurant study which included adolescent patients with FXS because of safety reasons.

### Analysis

#### CGI-I assessment and narratives

In both the core double-blind and open-label extension studies, global improvement, as a measure of treatment response, was assessed using the CGI-I scale [19]. The CGI-I assessments were carried out by the same rater across both the studies, whenever possible. The CGI-I score ranges from 1 to 7 (with 1 being “very much improved,” 4 being “no change,” and 7 being “very much worse”). The scoring was performed at weeks 2, 4, 8, and 12 in the core double-blind phase and at weeks 4, 12, and every 3 months thereafter in the open-label extension study.

Investigators who assigned patients a CGI-I score of 1 (very much improved), 2 (much improved), 6 (much worse), or 7 (very much worse) at any time during the studies were provided with a standard narrative template in which to describe further the changes observed in these patients. The CGI narratives were requested by a cutoff date on October 4, 2013. Narratives based on a score of 1 or 2 were considered trial responder narratives, whereas scores of 6 or 7 were considered trial worsening narratives. Patients receiving a score of 1 or 2 were subsequently referred to as “responders.”

A combination of inductive and deductive qualitative analysis was used to develop the coding scheme. Three Ph.D.-level experts in FXS independently reviewed responses on 10 randomly selected CGI narratives (five from each study) and identified all of the improved symptoms noted. These symptoms were then assigned to several broad categories for coding based on what is known about the FXS phenotype as well as on the nature of the written comments. If the narratives included any errors or discrepancies, they were not coded. Six broad categories (anxiety, behavior and mood, communication, cognitive/academic, engagement, and functional skills) were identified, comprising 14 subcategories of symptom improvement. The experts developed brief definitions for each category and agreed on the general criteria by which each would be assigned (see Table 1). Once the categories were identified, the coders independently reviewed all of the narratives and assigned each reported symptom to a category. Any disagreements in category assignment were discussed until a consensus was reached. The raters were blind to whether the narratives rated were from patients in the treatment group or the placebo group.

**Table 1:** Categories of improvement or worsening

1 Anxiety: any reference to reduction of anxiety, including OCD.
2. Behavior and mood: any reference to improvement in externalizing behaviors, including following directions, reduction in aggression, and complying with requests
a. Compliance—e.g., following direction, complying with requests
b. Transitions—e.g., willingness to change activities, ease of transitions
c. Externalizing behavior—e.g., aggression, SIB, destruction of property, temper tantrums
d. Mood—e.g., reference to changes in mood or irritability, listlessness
3. Communication: any reference to improvement in ability to communicate
a. Articulation—reference to intelligibility or speech production
b. Language—reference to ability to communication, word use, spontaneous use of speech (without a clear reference to social engagement/interactions)
4. Cognitive/academic: any reference to improvements in cognitive or academic skills
a. Cognitive—e.g., better scores on IQ test, ability to follow complex directions, increased cognition
b. Attention/memory/concentration—e.g., better focus, able to listen longer
c. Academic—e.g., better math skills, increased reading, better writing
5. Engagement: any reference to improvement in engagement with others and/or the environment
a. Social interactions—e.g., social interactions, social communication, engagement with others, willingness to engage
b. Engagement with environment—e.g., increased interests, engaging with activities
6. Functional skills: any reference to improvements in daily activities including self-care, independence with functional activities
a. Self-help/daily living skills—e.g., getting dressed, feeding self, toileting etc.
b. Independence/autonomy—e.g., improved work/job skills, more independence in household activities

*IQ* intelligence quotient, *OCD* obsessive compulsive disorder, *SIB* self-injurious behavior

### Statistical analysis

Any potential treatment effect was investigated using the following procedure: the number of patients expected to improve in a specific category was calculated under the assumption that each patient had the same chance to improve in that category (i.e., mavoglurant treatment would not make a difference). A difference between the observed and expected numbers of responders may indicate an effect of mavoglurant on a specific category. The strength of a potential effect was characterized using the Cochran–Mantel–Haenszel test to investigate trends according to the dose or a general association with treatment.

## Results

By the cutoff date, a total of 134 and 103 patients had reached 2 weeks (visit 4) or more of double-blind and open-label treatment, respectively. In the core double-blind study, 34 CGI-I scores of 1 or 2 were reported in 29 patients (i.e., 29 patients scored 1 or 2 at least once during the study up to the cutoff date). One patient had a CGI-I score of 6. There were no reports of patients with a CGI-I score of 7. All 35 narratives were received. There were 54 occurrences of CGI-I scores of 1 or 2 in 47 patients in the long-term extension study of which 52 narratives were received. One patient had a CGI-I score of 7 but this trial worsening narrative was not received. After duplicates/errors were removed, 29 and 47 narratives from the double-blind and open-label studies, respectively, were used for coding.

### Major categories and subcategorization

**Fig. 1:**
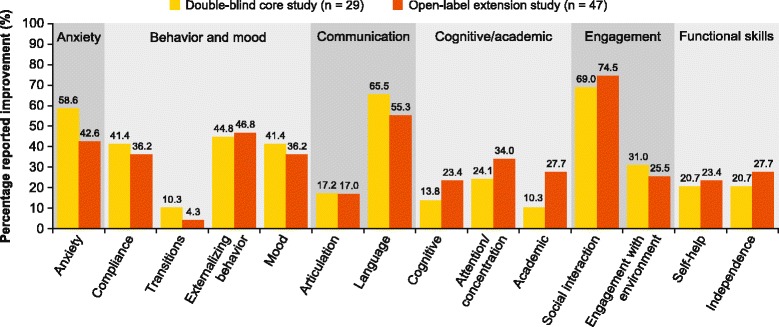
Percentage reported improvement across all subcategories

The distribution patterns of improvements reported across the subcategories were comparable between the core and long-term extension studies (Fig. 1). The most commonly reported subcategories in both the studies were social interaction, language, and externalizing. These results are presented as percentage of individuals (not subcategories) as one patient could improve in more than one subcategory.

**Fig. 2:**
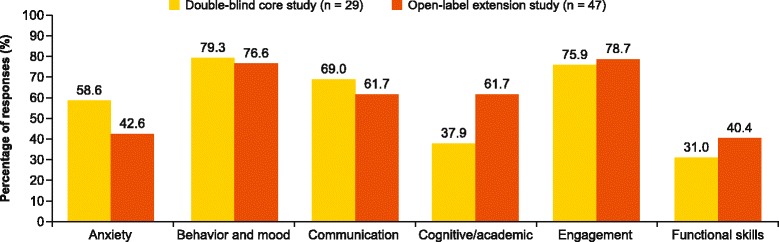
Percentage of patients who reported improvement in broad categories

Following consolidation of the subcategories into six broad categories, the reported improvements showed a similar distribution pattern in the blinded core and open-label extension studies. The results are based on the counts of individual patients (or number of responders) rather than the counts of CGI-I responses. The most frequently reported improvement categories were behavior and mood (79.3 %), engagement (75.9 %), and communication (69.0 %) in the core study (Fig. 2). In the long-term study, patients exhibited similar improvements in behavior and mood (76.6 %) and engagement (78.7 %) (Fig. 2).

In general, the percentage of patients improving was marginally lower in the long-term study, with the largest difference observed in anxiety (58.6 and 42.6 % in the core and long-term extension studies, respectively). However, the proportion of patients improving in the functional skills (40.4 vs 31.0 %) and cognitive/academic (61.7 vs 37.9 %) categories was greater (Fig. 2) in the long-term extension study compared with the core study.

### Distribution of responders in the double-blind core study according to treatment

After the unblinding of the core study, responders (patients receiving a score of 1 or 2) were analyzed according to their treatment group. This statistical analysis was conducted with 28 patients (as one patient of the 29 did not receive treatment). The CGI-I responders were similarly distributed across treatment arms (Table 2). Patients treated with mavoglurant did not show a higher prevalence of reported improvements than those treated with placebo. The response rate in each treatment group was also similar when the analysis was repeated for the six broad categories. Treatment with mavoglurant did not lead to a better outcome in any of these categories (Table 3). Worsening was reported in one patient on placebo with the following narratives: more aggression, noncompliant, more cursing at school, much harder to get out of bed, and lying on the ground at school.

**Table 2:** Distribution of responders across the treatment arms (double-blind core study)

	Placebo	Mavoglurant			Total
		25 mg	50 mg	100 mg	
Total number of patients (*N*)^a^	42	31	27	39	139
Number of patients who completed 12 weeks of treatment	40	31	27	37	135
Number of responders (observed number of patients who responded)	8	4	8	8	28
Expected number of responders^b^ (if distribution of response would have been random)	8.5	6.2	5.4	7.9	28

^a^Only data from 134 patients queried for CGI improvement

^b^Under the assumption of no treatment effect, i.e., identical response chance for each patient

**Table 3:** Distribution of responders in broad improvement categories across the treatment arms (double-blind core study)

	Placebo	Mavoglurant			*x* ^2^	d.f.	*p* value^a^
		25 mg	50 mg	100 mg			
Anxiety							
Number of responders	4	4	4	4	0.13/3.38	1/3	0.72/0.33
Expected number of responders	4.6	2.3	4.6	4.6			
Behavior and mood							
Number of responders	6	3	5	7	0.13/1.29	1/3	0.72/0.73
Expected number of responders	6	3	6	6			
Communication							
Number of responders	4	3	5	7	1.95/2.68	1/3	0.16/0.44
Expected number of responders	5.4	2.7	5.4	5.4			
Cognitive/academic							
Number of responders	3	2	1	5	0.31/4.26	1/3	0.58/0.24
Expected number of responders	3.1	1.6	3.1	3.1			
Engagement							
Number of responders	6	2	5	8	1.19/4.50	1/3	0.28/0.21
Expected number of responders	6	3	6	6			
Functional skills							
Number of responders	3	1	1	4	0.08/2.68	1/3	0.77/0.44
Expected number of responders	2.6	1.3	2.6	2.6			

Note: Expected number of responders is calculated under the assumption of equal response probabilities across treatments

^a^CMH test for equal response probabilities across treatments; total number of responders = 28; *x* ^2^ = chi-squared test statistic; d.f. = degrees of freedom; first *x* ^2^, d.f., and *p* value for trend, second for heterogeneity

## Discussion

Here, the qualitative assessment of change in overall symptom categories in adolescent patients with FXS, based on the narratives linked to the clinician-rated CGI-I scores, in a double-blind therapeutic study of mavoglurant and an open-label extension study is reported. Multiple anecdotes were received from the investigators during the double-blind treatment phase relating significant improvements in patients. Many of these anecdotes referred to categories such as language, cognition, and social interaction; categories that were not captured by the ABC-C, SRS, RBS, or CGI assessments used in the study. Consequently, a systematic collection of narratives for the patients rated by their clinician as “very much improved” or “much improved” (CGI-I score of 1 or 2) and “much worse” or “very much worse” (CGI-I score of 6 or 7) was instigated to further clarify the nature of these anecdotally reported improvements in the core double-blind and open-label extension studies.

In all, 29 of 134 patients in the core study and 47 out of 103 patients in the extension study scored 1, 2, 6, or 7 at least once. Only one patient from each study was rated as worsening (CGI-I score of 6 or 7). A key objective of this analysis was to better understand any potential efficacy of mavoglurant in patients with FXS by the analysis of symptom categories described in the clinician-provided narratives among patients with high levels of observed improvement. A further objective was to identify other categories of improvement with greater relevance in FXS than those currently included in the various outcome measurement scales.

Following treatment unblinding, responder distribution was assessed across the core study treatment arms. Individuals in the active treatment group did not perform better on any of the outcome categories compared with placebo. Overall, the CGI-I narrative analysis did not indicate an effect of mavoglurant on the number of responders or responses versus placebo. However, interestingly, cognitive/academic and functional gains were reported more frequently during the long-term extension study than during the core study. Benefits in these areas would be more expected to emerge with extended treatment duration, and the rater comments are likely to be based on a higher percentage of objective information (for example, school grades, standardized testing scores at school, ability to retain a job, and gaining toilet training skills) compared with the pure parental report for narratives in categories such as anxiety, engagement, and mood.

Furthermore, objective cognitive and functional measures were not directly assessed as a part of either study; thus, it is difficult to substantiate or refute the increased frequency in these areas of reported improvement with prolonged treatment in the open-label extension. This observation speaks to the need for novel trial designs in FXS and neurodevelopmental disabilities in general, which can evaluate long-term effects of treatments targeting underlying synaptic mechanisms and measuring disease modification in the form of improved learning and plasticity. However, these studies would likely require a time frame that is not amenable to placebo-controlled trials. Moreover, the need for a long time frame is likely to be particularly applicable to cohorts of adolescents and adults, in whom cognitive and functional changes are likely to evolve over more prolonged periods than for very young children.

Pre-clinically, many studies in *FMR1* knockout mice have shown behavioral and physiological FXS phenotypic rescue, such as spine morphology, prepulse inhibition, and audiogenic seizure corrections, following acute pharmacological inhibition of mGluRs [28–32]. In general, these corrections were more effective in younger animals [30]. Additionally, Michalon et al. demonstrated more correction of cognitive deficits with chronic versus short-term treatment with CTEP, a potent long-acting mGluR5 inhibitor, in young adult mice [33] further indicating the importance of long-term treatment. Although precise translation from pre-clinical studies into human trial duration can be debated, the chronic treatment duration reported (~5 weeks) in the mouse could potentially correlate to multiple years of treatment in a human. Thus, it is perhaps not surprising that cognitive gains (if any) might be relatively modest after only a year of open-label treatment. This also highlights the need for natural history models in FXS to determine the effects of drugs that may act over a very long term for which placebo-controlled trials are not possible.

Determination of efficacy or improvement through reliable, sensitive, standardized, and validated measures has been a major challenge for clinical trials in patients with FXS. Typical behavioral phenotype measures include ABC-C, CGI, SRS, VAS, and RBS, whereas cognitive end point measures include the Repeatable Battery for the Assessment of Neurological Status and VABS. However, there is a general lack of consensus over which end points are most useful and should be used to assess the potential treatment effect. This problem was the subject of a recent meeting arranged by the National Institutes of Health, aiming to identify and standardize outcome measures for potential use in clinical trials in patients with FXS. No single measure or set of measures was identified as optimal, but recommendations were made as guidelines [16].

The ABC-C is the most widely used assessment in clinical trials in FXS. It consists of five subscales, irritability, hyperactivity, lethargy/withdrawal, stereotypy, and inappropriate speech, associated with a 58-item checklist [34] and has been adapted for the community setting [35]. In 2012, Sansone et al. modified the scoring of the ABC-C for individuals with FXS resulting in addition of a sixth subscale, social avoidance [17], after completing a factor analysis on ABC assessments from approximately 600 subjects pooled from multiple FXS clinics, a finding subsequently replicated by Wheeler et al. [36]. Despite the modifications and widespread use of this scale in the FXS studies, there are certain limitations that remain. The ABC-C assessment is completed indirectly, by a parent or caregiver, potentially leading to unintentional bias and strong placebo effects [16]. Indeed, during the placebo run-in period of the core double-blind study, a strong placebo effect was observed in all treatment groups with substantial improvements in ABC-C_FX_ total scores.

Therefore, there is a need for more objective outcome measures. Although the CGI assessment is completed by a clinician, consideration is given to all available information, including caregiver anecdotes. Often the CGI ratings are predominantly based on the caregiver’s information and, as such, are subject to large placebo effects when there are no systematic objective data to incorporate into the clinician assessment. Furthermore, the CGI-I reports global changes of symptoms through a seven-point scale rating, and there are no scoring guidelines or anchors. Consequently, the measure may not comprehensively capture all the categories and can also be associated with variability. Anchors can help guide scoring and reduce variability. In addition, the CGI-I may simultaneously take into account the improvement in one area and worsening in another area and hence may hide a very specific area of response.

Another limitation of this study is the relatively small number of data points from the core study for analysis following unblinding with the expected number of responders in the subcategories being 6 or less per dosing group (Table 3). In some cases, there was a slight numerical advantage for the 100 mg BID mavoglurant-treated group, and it is possible that significance could not be demonstrated due to small sample size. In fact, a small but important effect size would not be detectable in this study.

Outcome measures should be sensitive enough to detect improvements over placebo responses and be reproducible and precise, so that they can capture most, if not all, of the symptom categories. Outcome measures that are based on direct assessment of the patient to quantify performance in language, functional, and cognitive categories are needed so as not to rely entirely on the caregiver’s reports of behavioral function and change, which appear to be highly variable in the FXS population and strongly susceptible to placebo effects.

## Conclusions

A method was established to capture symptom categories in FXS using the CGI-I narratives among patients with high levels of change during treatment in clinical trials with mavoglurant. This methodology and the categories identified could also be useful in future studies for capturing new items and indicating potential scoring anchors.

Analysis of the CGI-I narratives did not indicate a higher level of treatment response in patients treated with mavoglurant compared with patients receiving placebo. However, since many of the comments of specific improvements received from the investigators were based on parental report, this may not represent a completely objective alternative assessment. In addition, the number of responders was small, and therefore, the analysis may not be definitively conclusive about treatment response.

The lack of efficacy observed in the 12-week, double-blind, placebo-controlled study could be due to lack of efficacy of mavoglurant. However, this analysis does not rule out the possibility that efficacy could have been evident with other more robust measures for clinical trials in FXS such as cognitive or functional measures, as caregiver reported outcomes can be associated with a high placebo response. Future trials may require longer treatment duration, a younger study population with more plasticity, outcome measures focusing on improved function, cognition and/or academic achievement, or a modified trial design to evaluate “neural plasticity” through the incorporation of learning interventions, in order to allow efficacy to manifest.

## Abbreviations

ABC-C: Aberrant Behavior Checklist-Community Edition
CGI: Clinical Global Impression
CGI-E: Clinical Global Impression-Efficacy
CGI-I: Clinical Global Impression-Improvement
CGI-S: Clinical Global Impression-Severity
FXS: fragile X syndrome
mGluR1/5: metabotropic glutamate receptor subtypes 1 and 5
RBS-R: Repetitive Behavior Scale-Revised
SRS: Social Responsiveness Scale
SRS-A: Social Responsiveness Scale-Adult Research Version
VABS: Vineland Adaptive Behavior Scale
VAS: Visual Analog Scale
